# Effects of Absorption Kinetics on the Catabolism of Melatonin Released from CAP-Coated Mesoporous Silica Drug Delivery Vehicles

**DOI:** 10.3390/pharmaceutics13091436

**Published:** 2021-09-09

**Authors:** Irene Moroni, Alfonso E. Garcia-Bennett

**Affiliations:** 1 Department of Molecular Sciences, Macquarie University, Sydney, NSW 2109, Australia; irene.moroni@mq.edu.au; 2 ARC Training Centre for Facilitated Advancement of Australia’s Bioactives (FAAB), Macquarie University, Sydney, NSW 2109, Australia

**Keywords:** melatonin, drug delivery, mesoporous silica, catabolism, bioavailability

## Abstract

Melatonin (MLT) is a pineal hormone involved in the regulation of the sleep/wake cycle. The efficacy of exogenous MLT for the treatment of circadian and sleep disorders is variable due to a strong liver metabolism effect. In this work, MLT is encapsulated in mesoporous silica (AMS-6) with a loading capacity of 28.8 wt%, and the mesopores are blocked using a coating of cellulose acetate phthalate (CAP) at 1:1 and 1:2 AMS-6/MLT:CAP ratios. The release kinetics of MLT from the formulations is studied in simulated gastrointestinal fluids. The permeability of the MLT released from the formulations and its 6-hydroxylation are studied in an in vitro model of the intestinal tract (Caco-2 cells monolayer). The release of MLT from AMS-6/MLT:CAP 1:2 is significantly delayed in acidic environments up to 40 min, while remaining unaffected in neutral environments. The presence of CAP decreases the absorption of melatonin and increases its catabolism into 6-hydroxylation by the cytochrome P450 enzyme CYP1A2. The simple confinement of melatonin into AMS-6 pores slightly affects the permeability and significantly decreases melatonin 6-hydroxylation. Measurable amounts of silicon in the basolateral side of the Caco-2 cell monolayer might suggest the dissolution of AMS-6 during the experiment.

## 1. Introduction

In humans the hormone melatonin is primarily endogenously produced at night-time in the pineal gland and regulated by the suprachiasmatic nuclei from external light stimulation [1,2,3]. Clinical studies have correlated the rhythmic production of melatonin to the sleep/wake cycle, leading to identifying melatonin as an internal sleep promoter [4,5]. The administration of exogenous melatonin is commonly used in the treatment of circadian and sleep disorders as a chronobiotic therapy [6,7]. Melatonin is commercially available worldwide in a wide range of formulations and doses that vary from 1.99 mg (REMfresh^®^ [8]) to 20 mg (Nutricology^®^). Melatonin can be purchased both as over-the-counter products and with a medical prescription, but its efficacy for treating these sleep disorders is variable [9,10,11]. The reasons for this variability have not been fully identified, but variations in melatonin absorption through the gastrointestinal (GI) barrier when administered orally and a strong first-pass metabolism of melatonin play key roles [12,13]. 

The catabolism of melatonin occurs via both enzymatic and nonenzymatic pathways [14]. Hepatic catabolism in humans is dominated by cytochrome P450 enzymes (CYP), leading to the formation of 6-hydroxymelatonin, where CYP1A2 is the most prevalent enzymatic catalyst. Other CYP450s involved, to a lesser extent, in the enzymatic degradation of melatonin into 6-hydroxymelatonin are CYP2C9 and CYP2C19 [15]. The conjugation of 6-hydroxymelatonin with sulphuric acid or glucuronic acid, followed by excretion in urine, results in a significant lowering of the bioavailability of the exogenous dose [16]. The nonenzymatic pathway for the 6-hydroxylation of melatonin involves consecutive reactions with free radicals, such as hydroxyl radicals (•OH), and represents a minor catabolic route [14].

The bioavailability of oral melatonin ranges between 9 and 33% in humans, with a time to maximum concentration (T_max_) of approximately 50 min following oral immediate-release formulations of melatonin [17]. Formulations that promote the release of melatonin in selected sections of the gastrointestinal tract where the greatest absorption of melatonin occurs, such as the in the ileum [18], might enhance the absorption of melatonin. However, it is not clear if this translates to improvements in the bioavailability given the propensity of melatonin to be metabolized into 6-hydroxy melatonin by CYP enzymes.

Mesoporous silica particles consisting of amorphous silica walls and pores in the mesoscale range (2–50 nm) are a versatile family of biocompatible drug carriers [19,20,21,22] with the ability to host and control the release of drugs from within their pores [23,24,25,26]. The generic mechanism for the release of drugs from mesoporous silica involves a diffusion-controlled but spatially hindered transport of the drug from the internal pore space to the exterior of the particle [27]. This can be further controlled by altering any electrostatic interactions between the silica surface and the adsorbed molecules or by modifying the particle’s surface with the addition of nanoelements to obtain stimuli-responsive systems, such as magnetic nanoparticles or enteric polymers [28,29]. A large number of silica mesoporous structures have been investigated as formulation aids, but the material known as AMS-6 (Anionic Mesoporous Silica-6) was chosen in this work, because it possesses a spherical particle morphology with an average particle size distribution of approximately 250 nm [30]; this size prevents the particles from crossing the mucus layer and, subsequently, the epithelial cells of the intestinal barrier. The AMS-6 particles also possess three dimensionally connected pores that offer a rapid diffusion of drugs from within the pores in comparison to two-dimensional connected porous structures. 

**Figure 1:**
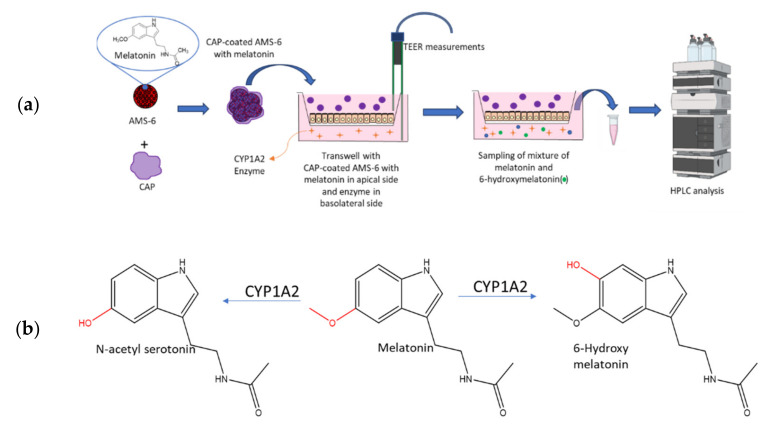
(**a**) Schematic representation of the formulation studied in this work and the Caco-2 cell model utilised to determine the effect of the release rate on the metabolism of melatonin from the formulation. Melatonin was loaded into mesoporous silica via a wetness impregnation of the hormone, using ethanol as the solvent, followed by evaporation using a rotary evaporator. A similar process was used to coat mesoporous silica with CAP. See the Materials and Methods Section 2.3 and Section 2.4. (**b**) Main products of the catabolism of melatonin in the liver of vertebrates by cytochrome P450 enzymes (CYP) such as CYP1A1, CYP1A2, CYP1B2 or CYP2C19 [19].

Control of the drug release rate, influencing the absorption rate of melatonin within a specific location of the gastrointestinal tract, may affect the hepatic metabolism of melatonin. In fact, a sudden burst release of the drug within the small intestine can saturate hepatic enzymes without using a higher dose and, consequently, increase the percentage of melatonin that reaches systemic circulation. To investigate this, a Caco-2 monolayer cell model was used as an intestinal epithelial barrier to determine the effects of formulations of mesoporous silica loaded with melatonin. The Caco-2 cell line is derived from heterogeneous human epithelial colorectal adenocarcinoma cells and, when cultured, appropriately expresses a phenotype similar to that of small intestine enterocytes [31,32,33]. In fact, when cultured in a monolayer, Caco-2 cells exhibit tight junctions and microvilli typical of small intestine cells [32]. Drug absorption experiments using Caco-2 monolayers show good correlation with absorption experiments carried out on a part of the jejunum [34,35]. Measurements of the transepithelial/transendothelial electrical resistance (TEER) allow to evaluate the Caco-2 cell monolayer integrity grown on a Transwell membrane support. The Caco-2 cell monolayer model is often used to measure permeability changes between pure and formulated drugs. A recent example is the study by Raza et al. [36], in which the permeability of meropenem through a Caco-2 monolayer was enhanced using a drug delivery system containing mesoporous silica. In this work, to delay the release of melatonin from the mesopores, cellulose acetate phthalate (CAP) was coated as a layer on the external surface of the particles. CAP is often used as an enteric coating [37] to avoid the release of APIs in the acidic environment of the stomach. The combination of CAP and mesoporous silica has been used before in a study by Garcia-Casas et al. [38], in which antioxidants and CAP were deposited on the surface of mesoporous silica particles to achieve a delayed release of antioxidants in gastric media. The permeability of melatonin through the monolayer was measured using high-performance liquid chromatography (HPLC) [39] (Figure 1). Subsequently, the hydroxylation of melatonin by the CYP1A2 enzyme was investigated to determine any correlation between the release rate from the drug delivery vehicles and the catabolic rate. The transport of molecules across the intestinal barrier can occur via paracellular diffusion through tight junctions (between epithelial cells) or transcellular transport via endocytosis/exocytosis, which can be mediated by membrane receptors. Paracellular transport involves molecules with a molecular weight <600 Da, and paracellular permeability is controlled by the pore size or the tight junctions that are usually in the range of 8 to 9 Å. The transcellular transport is a less restrictive pathway that involves the transport of larger molecules or particles [40].

## 2. Materials and Methods

### 2.1. Materials

Unless stated, all chemicals were purchased from Sigma-Aldrich (Merck Pty. Ltd., Castle Hill, NSW, Australia) and used without further purification. Test compounds and formulations were denominated in the following way: free melatonin—MLT, mesoporous silica particles—AMS-6, mesoporous silica containing melatonin—AMS-6/MLT and mesoporous silica containing melatonin coated with cellulose acetate phthalate AMS-6/MLT:CAP (ratios 1:1 or 1:2). The CAP utilised in this study had a molecular weight of 2534.12 g/mol.

### 2.2. Synthesis Methods

The synthesis of AMS-6 was conducted following a published procedure [41]. N-Lauroyl-L-alanine (product number 61726, 1.25 g) was dissolved in water (250 mL, Milli-Q) in a PVC bottle by leaving the mixture overnight at 80 °C. Subsequently, the mixture was stirred at 1000 rpm at 80 °C for 10 min. Under the same stirring and heating conditions, the co-structure-directing agent (3-aminopropyl) triethoxysilane (APES, product number 440140, 1.25 g) was added, followed immediately after by the silica source tetraethyl orthosilicate (TEOS, product number 131903, 6.25 g). The resulting mixture was stirred at 1000 rpm at 80 °C for 1 h; then, the stirring rate was reduced to 500 rpm. After 24 h, the stirring was stopped, and the mixture was left at RT overnight. The product was recovered by filtration, and then, the surfactant was extracted by redispersing the product of the reaction into a mixture of ethanol (EtOH, product number 443611):hydrochloric acid (HCl, product number 320331, 37%) 80:20 and stirring at 80 °C for 4 h. The extracted product was recovered by filtration and calcined in the air at 550 °C for 3 h.

### 2.3. Melatonin Loading Procedure

One gram of AMS-6 was dispersed into 200 mL of ethanol. Separately, 310 mg of melatonin was added to 50 mL of ethanol and sonicated for 30 min. The two mixtures were combined and stirred at RT with a magnetic stirrer at 500 rpm for 10 min. The solvent was then evaporated using a rotary evaporator (International Scientific Group Ltd., Downham Market, UK) connected to a programmable pump (PC 3000 Series, Vacuubrand GMBH + CO KG, Wertheim, Germany). The pressure was progressively decreased using the following program: 10 min at 450 mbar, 10 min at 400 mbar, 10 min at 350 mbar, 10 min at 300 mbar, 10 min at 250 mbar, 20 min at 200 mbar, 20 min at 150 mbar, 20 at 100 mbar, 20 min at 50 mbar and 20 min at 1 mbar. The temperature of the bath was set at 45 °C, and the stirring rate was 20 rpm. The sample obtained was called AMS-6/MLT.

### 2.4. CAP Coating Procedure

One hundred and eighty milligrams of AMS-6/MLT were dissolved into 150 mL of acetone, and separately, 180 mg of CAP was dispersed into 150 mL of acetone. The two mixtures were sonicated for 30 min, then mixed together in a 1-L flask and stirred at 500 rpm at RT for 10 min. The acetone solvent was then evaporated using a rotary evaporator with decreasing pressure using the following program: 10 min at 700 mbar, 10 min at 600 mbar, 20 min at 550, 20 min at 500, 20 min at 450 mbar, 20 min at 400 mbar, 20 min at 350 mbar, 20 min at 300 mbar, 20 min at 250 mbar, 20 min at 200 mbar and 60 min at 1 mbar. The temperature of the water bath was kept at 35 °C, and the stirring rate was 20 rpm throughout. The sample resulting from this coating procedure was called AMS-6/MLT:CAP(1:1). To obtain AMS-6/MLT:CAP(1:2), the same procedure was used, but the amount of CAP was doubled. 

### 2.5. Structural and Textural Characterization

Powder X-ray Diffraction was performed on samples of calcined mesoporous silica AMS-6, MLT and AMS-6/MLT to assess the mesoscale order of the pores and the crystallinity of melatonin. A Bruker D8 Discover diffractometer (Bruker, Preston, VIC, Australia) was used with a Cu-Kα radiation as the X-Ray source (λ = 1.54 Å). The diffraction patterns were collected between 0.5° and 30° in 2θ. The data was collected and analysed with DIFFRAC.SUITE™ software from Brucker.

Nitrogen porosimetry was used to measure the pore size distribution, pore volume and surface area of all the samples. Measurements were performed with a Micromeritics TriStar II Surface Area and Porosity instrument (Micromeritics Instrument Corporation, GA, USA). Data were collected and analysed using TriStar II 3020 software. Prior to the adsorption isotherm measurements, the samples were degassed using a Micromeritics VacPrep 061 Degass System at 100 °C (AMS-6) or at RT (AMS-6/MLT and AMS-6/MLT:CAP samples) for 12 h. The surface area was calculated using the BET equation in a relative pressure range between 0.05 and 0.2 [42]. The total pore volume was calculated from the amount of gas adsorbed at P/P^o^ = 0.95. The pore size distribution curves were derived using the density functional theory, assuming a cylindrical pore model for all the samples [43]. 

Scanning electron microscopy (SEM) was used to determine the morphology, surface characteristics and size of AMS-6 using a JSM-7401F Field Emission SEM (JEOL Ltd., Tokyo, Japan) operating at 1 to 2 kV with no gold coating, using the gentle beam mode at magnifications between 5 and 50,000. 

A thermogravimetric analysis was used to calculate the amount of drug loading and surface coating using a Thermogravimetric Analyser TGA-2050 from TA Instruments (DE, USA). The derivative weight loss calculation was performed using TA instruments software (TA Instruments, Universal analysis 2000, version 3.0 G). The mass of every sample was between 2 and 5 mg, and the temperature increased from room temperature to 800 °C with a heating rate of 20 °C/min between room temperature and 110 °C and a rate of 10 °C/min between 110 °C and 800 °C, with an argon flow rate of 50 mL/min.

Differential Scanning Calorimetry (DSC) was measured using a Netsch STA 449 F3 Jupiter instrument to determine the crystallinity of the loaded MLT. An argon flow of 20 mL/min was used. The temperature was increased from 20 °C to 200 °C (heating rate of 10 °C/min), then maintained at 200 °C for 15 min; subsequently, the temperature was decreased to 20 °C (cooling rate = 10 °C/min) and maintained at 20 °C for 15 min; a second heating/cooling run was performed in the same way.

A Fourier-Transform Infrared Spectroscopy (FTIR) analysis was carried out to confirm the sample composition of the polymer-coated silica materials using a Nicolet iS5 FTIR Spectrometer with iD5 ATR accessory featuring a laminated diamond crystal (Thermo Scientific, MA, USA). The samples were analysed without any dilution.

Dynamic light Scattering (DLS) experiments were performed with a Zetasizer ZS (Malvern Instruments, Worcestershire, UK) with a 173° detector angle at 25 °C with a He-Ne laser (633 nm, 4-mW output power) as the light source. A dispersion of AMS-6 particles in filtered Milli-Q water (20 µL, 1 mg/mL) was prepared and filled into a disposable folded capillary cell (DTS1070) (Malvern Instruments). 

Inductively Coupled Plasma Mass Spectrometry (Q-ICP-MC) was used to determine the concentration of silicon in the basolateral solution of the Caco-2 cell monolayer after the permeability experiments. The measurements were carried out using an Agilent 7500 quadrupole Inductively Coupled Plasma Mass Spectrometer (Q-ICP-MC) at Macquarie GeoAnalytical (MQGA, Agilent Technologies, Santa Clara, CA, USA), Macquarie University. The samples in HBSS (Gibco, product number 14025092) were diluted using 2% HNO_3_ (product number 438073) with a dilution factor of 100. The calibration standards were prepared by spiking 1% HBSS solution with 0.5, 1, 2, 5, 10, 50, 100 and 200 ugL^−1^ of silicon. 

### 2.6. Dissolution Measurements of Melatonin-Loaded Formulations

The release measurements were conducted in a USP Type 2 Dissolution Apparatus (paddles) 708-DS coupled with a Cary 60 UV-Vis spectrophotometer (Agilent Technologies, CA, USA) by loading the different formulations (dose of MLT equivalent to 16 mg for all formulations) into rapidly dissolving cellulose capsules. The measurements were conducted at 37 °C with a stirring rate of 50 rpm in simulated gastric fluids (SGF, pH 1.2) and simulated intestinal fluids (SIF, pH 6.8). The SGF media was prepared by adding sodium chloride (NaCl, product number S9888, 2.0 g/L) and hydrochloric acid 37% (HCl, product number 320331, 1.5% *v*/*v*) in Milli-Q water. The SIF media was prepared by dissolving sodium hydroxide (NaOH, product number S8045, 0.896 g/L) and potassium phosphate monobasic (KH_2_PO_4_, product number P8709, 6.805 g/L) in Milli-Q water.

### 2.7. Caco-2 Cell Culture and Formation of Monolayer on the Permeable Supports

The cells were preserved in liquid nitrogen, and 1 mL of culture medium containing the cells was mixed with 90% foetal bovine serum (FBS, ThermoFisher, product number 10099133) and 10% dimethyl sulfoxide (DMSO, product number D2650). When thawed, the cells were mixed with 9 mL of culture medium. The culture medium was Dulbecco’s modified Eagle’s medium (DMEM, ThermoFisher, product number 11960-044) high glucose with L-glutamine (product number G8540), 10% FBS, 1% nonessential amino acids (Gibco, product number 11140-050) and 1% penicillin–streptomycin solution (PEST, Gibco, product number 15140-122). The cells were centrifuged at 300 rpm for 5 min, the supernatant was removed and cells were redispersed in 3 mL of culture medium. The cells were transferred to a 25-cm^2^ flask (1 mL of cells in the cell culture medium plus 12 mL of fresh cell culture medium) and incubated at 37° C. The cell culture medium was changed every 2 days, and the cells were passaged once a week.

The protocol employed to form Caco-2 monolayers has been reported previously [44]. Briefly, after culturing the cells in flasks and passaging them at least twice (passage of cells used for the final experiment: 30–32), the cells were transferred onto permeable supports (Corning Transwell polyester membrane cell culture inserts, 12-mm Transwell with 0.4-µm pore polyester membrane insert, tissue culture-treated and sterile) as follows. The cell culture medium was removed from the flask, and the cells were washed with 5-mL phosphate-buffered saline (PBS). After removing the PBS (ThermoFisher, product number 10010023), 3 mL of TryplE reagent (ThermoFisher, product number 12604013) were added to the flask, and the cells were incubated at 37 °C for 10–15 min. The detached cells were added to 7 mL of culture medium and centrifuged at 300 rpm for 5 min. The supernatant was then removed, and the cells were dispersed into 6 mL of culture medium. The permeable supports were prewetted with about 0.1 mL of medium for at least 2 min before seeding the cells. To seed the cells onto the supports, 0.5 mL of medium with the resuspended cell solution was added to each filter (the apical side of the Transwell). The basolateral side of the Transwell was then filled with 1.5 mL of fresh medium. The Transwell plate was incubated at 37 °C for 6 h. The medium on the apical side was then removed and replaced with 0.5 mL of fresh medium to avoid the formation of multilayers. To maintain the cells, the medium was changed every second day in the following way: the medium from the basolateral side was aspirated first, followed by careful removal of the medium from the apical. It was critical not to touch the supports with the tip of the pipettes to avoid damaging the integrity of the monolayer. A fresh medium was then used to fill the apical side (0.5 mL) first and, subsequently, the basolateral side (1.5 mL). 

### 2.8. Permeability Studies

Permeability studies were performed after growing the Caco-2 monolayer for 21 days, as previously reported [44]. The first step involved the preparation of the apical and basolateral solutions. The apical solution consisted of Hank’s balanced salt solution (HBSS) without phenol red and a MES buffer (product number M1317, 1 mL of MES in 99 mL of HBSS). The basolateral solution consisted of HBSS without phenol red with the addition of 4-(2-hydroxyethyl) piperazine-1-ethane sulfonic acid (HEPES, product number H0887, 1 mL of HEPES in 99 mL of HBSS). The solutions were warmed to 37 °C, and the medium was removed from the Transwell. The supports were washed by adding HBSS and incubating at 37 °C for 15–20 min. The transepithelial/transendothelial electrical resistance (TEER) was measured (t_0_). The average initial TEER value was 823 ± 19 Ω. The HBSS was then removed from the Transwell. Melatonin or melatonin-loaded formulation was dispersed into the apical solution (at an equivalent melatonin concentration of 50 µM), and 0.5 mL of it was added to each support. The basolateral solution was then added to the basolateral side (1.5 mL) of each Transwell. Melatonin and each melatonin-loaded formulation was added to 3 different Transwells to obtain statistically significant results. The Transwell plates were incubated at 37 °C on a shaker at 500 rpm. The TEER was measured every 30 min, and aliquots (250 µL) from the basolateral side of the Transwell were sampled every 15 min for 3 h, and every time, they were replaced by fresh basolateral solution (250 µL). To the samples was then added 250 µL of Milli-Q water to increase their volume, filtered (nylon filters, 4 mm in diameter, 0.2-µm pore size, Bio-Strategy) and then analysed with high-performance liquid chromatography (HPLC) to measure the concentration of melatonin.

### 2.9. Catabolism of Melatonin with CYP1A2 Enzyme

The same apical and basolateral solutions used for the permeability study were prepared to assess the hydroxylation of melatonin. The medium was removed from the Transwell. The supports containing the cell monolayer were washed by adding HBSS solution and incubating at 37 °C for 15–20 min, and the HBSS solution was removed prior to the addition of the apical and basolateral solutions to the Transwell. The TEER was measured (t_0_). Melatonin or melatonin-loaded formulations were dispersed into 0.5 mL of apical solution (concentration of melatonin: 50 µM) and added to each support. The CYP1A2 enzyme was dispersed into 1.5 mL of basolateral solution (concentration of 3 µg/mL), and the solution was added to the basolateral side of each Transwell. Each experiment was repeated 3 times in different Transwells to obtain statistically significant results. The Transwell plates were incubated at 37 °C on a shaker at 500 rpm. The TEER was measured every 30 min, and the basolateral solution was recovered at specific time points (1, 2 and 3 h). The enzymatic reaction was stopped by adding 0.5 mL of ethyl acetate and mixing. For each sample, the ethyl acetate layer was removed, and the water layer was filtered (nylon filters, 4 mm in diameter, 0.2-µm pore size, Bio-Strategy). The samples were analysed with HPLC to measure the concentration of 6-hydroxymelatonin and remaining melatonin simultaneously with high-performance liquid chromatography (HPLC). An Agilent 1260 Infinity system with a reversed-phase C18 column (5 µm, 4.6 × 250 mm; Grace Davison Discovery Sciences, Rowville, Vic, Australia) and a UV detector were used. The mobile phase consisted of water:acetonitrile (product number 34851) (60:40) run at a flow rate of 1 mL/min. The analysis time for each sample was 6 min. The column and mobile phase were kept at 40 °C, and the injection volume of each sample was 10 µL. The wavelength used for UV detection was 279.0 nm. The samples were analysed in triplicates. The standard curve for both melatonin and 6-hydroxymelatonin was prepared in water, and the standard concentrations varied between 0.1 µg/mL and 2 µg/mL.

## 3. Results

### 3.1. Physicochemical Characterisation of Formulations

**Figure 2:**
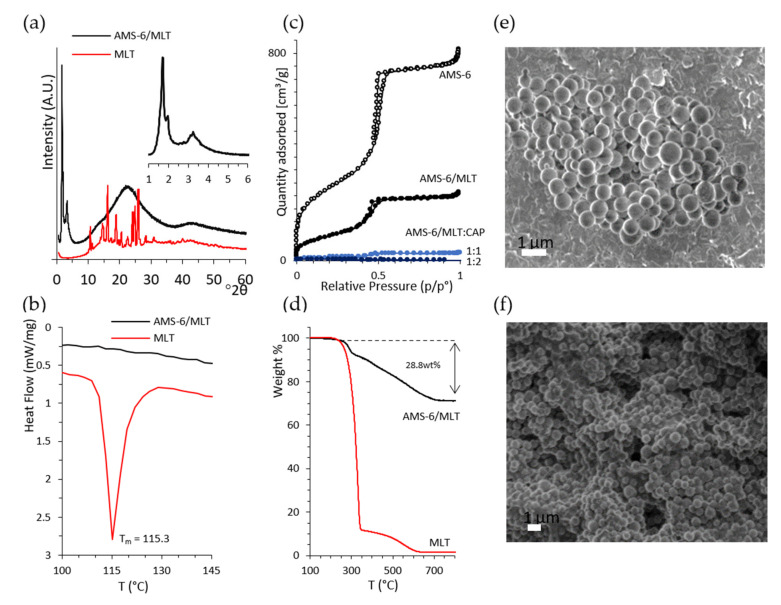
(**a**) X-ray diffractogram of the pure melatonin (MLT) and melatonin loaded within mesoporous silica (AMS-6/MLT), showing the absence of diffraction peaks for crystalline MLT after loading. The inset shows the low-angle mesoscale peaks typical of the cubic structure of AMS-6 [30]. The cubic unit cell was calculated to be 126.6 Å. (**b**) The DSC trace of MLT shows a melting endotherm peak at 115.3 °C, which is absent in the trace of AMS-6/MLT. (**c**) Nitrogen sorption isotherm curves from AMS-6, AMS-6/MLT and AMS-6/MLT:CAP-coated samples showing the decrease in porosity parameters as a function of MLT loading and CAP coating. (**d**) Thermogravimetric analysis confirming a loading of 28.8 wt% of MLT within the pores. Representative SEM images recorded on (**e**) AMS-6 and (**f**) AMS-6/MLT:CAP(1:1).

Melatonin (MLT) was loaded into mesoporous silica AMS-6 via wetness impregnation at a loading amount of 30 wt%. The average particle size of AMS-6 from the DLS analysis was measured to be 246.7 ± 62.6 nm (Figure S1a, Supplementary Material). The characteristic X-ray diffraction peaks of pure crystalline MLT (Figure 2a) between 10 and 40° 2θ degrees were not present in the loaded AMS-6/MLT formulation sample, which only showed the mesoscale diffraction peaks owing to the bi-continuous cubic arrangement of the pores of AMS-6 [30,45]. The absence of diffraction peaks suggested the loading of MLT within the mesopores in an amorphous state, which was confirmed by the absence of a melting endotherm peak in the DSC traces recorded for AMS-6/MLT (Figure 2b). The nitrogen adsorption isotherm data showed a significant decrease in the pore size distribution (54.9Å to 50.1 Å) mesopore volume (1.17 cm^3^/g to 0.38 cm^3^/g) and surface area (1003.3 m^2^/g to 355.4 m^2^/g) of AMS-6 as a function of MLT loading, indicating that the drug resided within the mesopores (Figure 2c). The actual final loading in AMS-6/MLT was confirmed by the thermogravimetric analysis to be 28.8 wt% (Figure 2d).

The TGA curves of CAP-coated formulations is shown in the Supporting Information (Figure S1b). Further functionalisation of AMS-6/MLT with CAP fills and blocks the mesopores of AMS-6 almost to completion, with measured mesopore volumes for AMS-6/MLT:CAP(1:1) and AMS-6/MLT:CAP(1:2) below 0.01 cm3/g. The adsorption step assigned to intraparticle adsorption at relative pressures > 0.95 is absent in the adsorption isotherms of the CAP functionalised particles, indicating the presence of CAP in these intraparticle spaces. SEM images of AMS-6 and AMS-6/MLT:CAP(1:1) are shown in Figure 2e,f and reveal the presence of CAP as a coating embedded in the particles. The CAP coating covers agglomerates of particles and fills all the gaps between the particles; consequently, the texture of the CAP-coated sample appears to be very different from the uncoated one.

**Figure 3:**
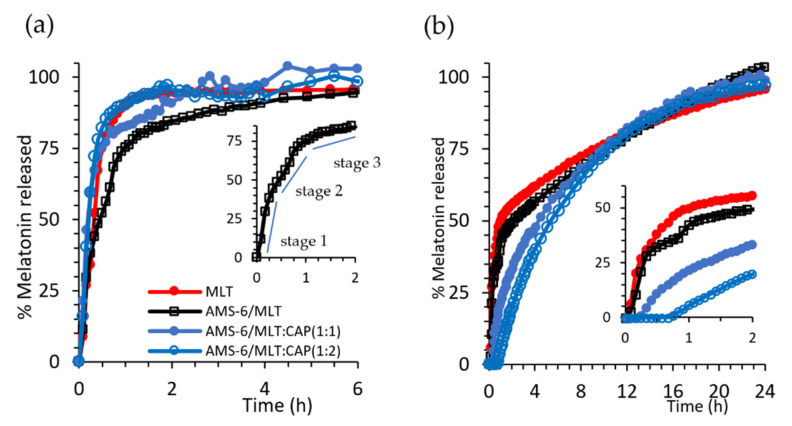
(**a**) Kinetic release measurements of MLT and test formulations in SIF (pH 6.8) and (**b**) SGF (pH 1.2). The inset in (**a**,**b**) shows the region 0 < t < 2 h for the selected formulations. For ease of comparison, error bars were omitted. The same figure with error bars can be found in the Supplemenatry Material (Figure S2). Every data point represents the average value obtained from a triplicate sample, and the error bars shown in Figure S2 correspond to the calculated standard deviation.

Analysis by FTIR (Figure 3 and Figure S1b) shows signature vibrational peaks for siloxane linkages in the silica wall (*v*Si-O-Si, 1068 cm^−1^), for the carbonyl group in MLT (*v*C=O, 1623 cm^−1^) or the carbonyl group of CAP (*v*C=O, 1720 cm^−1^). The carbonyl group of MLT is visible in the spectra of AMS-6/MLT. The siloxane peak of AMS-6 and the hydroxyl peak of CAP [46] (*v*OH, 1030 cm^−1^) merge into one single peak in AMS-6/MLT:CAP(1:1). Two strong bands at 3290 and 3302 cm^−1^ are visible in the spectrum of MLT, which can be assigned to the N-H stretching vibrations of MLT [47]. These are also visible in the spectra of AMS-6/MLT and AMS-6/MLT:CAP(1:1), albeit with a small shift to higher frequencies. There is no proof of interaction between the different components of the formulation.

### 3.2. Kinetic Release Measurements of MLT-Loaded Formulations

Dissolution experiments were conducted under sink conditions in SIF (pH 6.8) and SGF (pH 1.2) to assess the release kinetics of MLT as a free drug and in the prepared formulations (Figure 3). Pure MLT dissolves rapidly in SIF, with 93% of the drug dissolved within 1 h. A release from AMS-6 occurs with a three-stage profile (Figure 3a inset), whereby MLT is fist released rapidly as the drug close to the pore surface dissolves at a similar dissolution rate to pure MLT (stage 1, 0–0.5 h). This is followed by a release from the internal pore structure, which occurs at a slower rate (stage 2, 0.5–1 h), and finally, the MLT molecules occluded within the internal pores are released (stage 3, >1 h) at an even slower rate. At a neutral pH, the dissolution of CAP is fast, offering no diffusion barrier for MLT dissolution [48]. However, a faster release rate is observed for CAP-coated AMS-6 particles in SIF than for AMS-6/MLT alone. This suggests that some MLT is displaced to the exterior of the particle during the CAP loading procedure and remains trapped in the polymer coating around the particles.

The kinetic release curves in SGF differ due to the pH-dependent solubility of MLT and CAP. Pure MLT dissolves at a slower rate in SGF. Only 50% of that added in the dissolution bath was dissolved within 1 h, and a slower rate of dissolution was observed thereafter (Figure 3b).

The loading of MLT within AMS-6 does not affect the dissolution, which follows the same profile as pure MLT. Coating AMS-6/MLT with CAP results in a time lag for the dissolution of MLT, which increases to over 30 min for AMS-6/MLT:CAP(1:1). The delay in the release does not prevent either of the CAP-coated formulations from reaching 100% release of the drug in SGF. These results are consistent with the well-known enteric coating properties of CAP [48]. Kinetic release constants for the tested formulations were calculated, assuming the rate of dissolution of MLT was faster than the rate of diffusion, i.e., a diffusion-dependent model or Higuchi model [49], with a constant kH (Table 1).

Apart from the aforementioned differences with respect to pH, the rate constants indicated a 1.7-fold decrease in the release kinetics for AMS-6/MLT:CAP(1:2) compared to AMS-6/MLT. Coating with higher amounts of CAP does not alter the release kinetics of MLT, confirming the role of CAP as a protective coating for the release of MLT from mesoporous silica particles.

### 3.3. Permeability Studies

**Figure 4:**
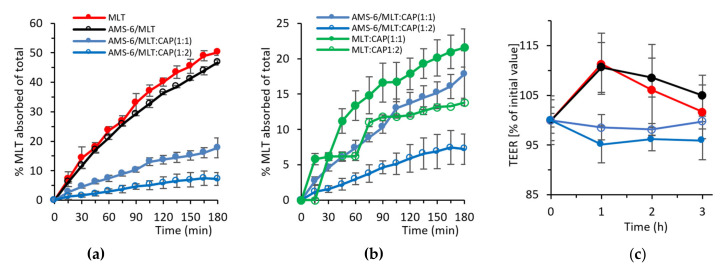
Absorption kinetics of MLT as a molar percentage of the total added across a Caco-2 monolayer for (**a**) MLT, AMS-6/MLT, AMS-6/MLT:CAP(1:1) and AMS-6/MLT:CAP(1:2) and (**b**) CAP-coated MLT in the absence of AMS-6 mesoporous silica. (**c**) TEER measurements showing the Caco-2 monolayer integrity during the absorption experiments. Every data point represents the average value obtained from a triplicate sample, and the error bars correspond to the calculated standard deviation.

Permeability studies of the test MLT formulations through a Caco-2 monolayer were performed as described in Section 2.6 at pH 7.4. The amount of MLT absorbed across to the basolateral side of the Transwell was measured over a period of 3 h (Figure 4a). Measuring for a period of 3 h was considered to be enough, as, in that time, the CAP-coated formulations released 100% of MLT and pure MLT and AMS-6/MLT released more than 85% of MLT. The apparent permeability coefficient (Papp) was calculated using the following equation:

P_app_ = V_r_ x (dC)/(dt) × 1/AC_0_ (1)

where Vr is the volume of the recipient compartment, dC/dt is the slope of the cumulative concentration of MLT in the recipient chamber over time, A is the membrane surface area and C0 is the MLT initial concentration on the basolateral side [50]. The encapsulation of MLT within AMS-6 decreased its permeability coefficient from 3.08 × 10^−5^ to 2.81 × 10^−5^ cm s^−1^. Coating with CAP significantly decreased the permeability of MLT, with coefficients of 1.06 × 10^−5^ and 4.81 × 10^−6^ cm s^−1^ calculated for AMS-6/MLT:CAP(1:1) and AMS-6/MLT:CAP(1:2), respectively. Given that the dissolution profiles measured in SIF (Figure 3b) showed only small differences in the release rate of CAP-coated and uncoated AMS-6/MLT, the differences in the permeability coefficient may be attributed to the absence of sink conditions in the permeability study. The lack of sink conditions may change the dissolution profiles of CAP-coated and uncoated AMS-6/MLT, therefore varying the permeability rates. To explore this further, permeability studies were also conducted on formulations of MLT with CAP in the absence of AMS-6 mesoporous silica (Figure 4b). These results showed that there was a decrease in permeation with the combination of AMS-6 and CAP. The measurements of TEER showed an initial decrease in the monolayer integrity of the cells in AMS-6/MLT:CAP 1:1 and 1:2 in comparison to the MLT-incubated monolayers (Figure 4c). However, this change in the monolayer integrity was not significant after 2 h. Overall, the integrity of the Caco-2 monolayer did not appear to be disrupted by the formulations.

### 3.4. Hydroxylation of MLT

**Figure 5:**
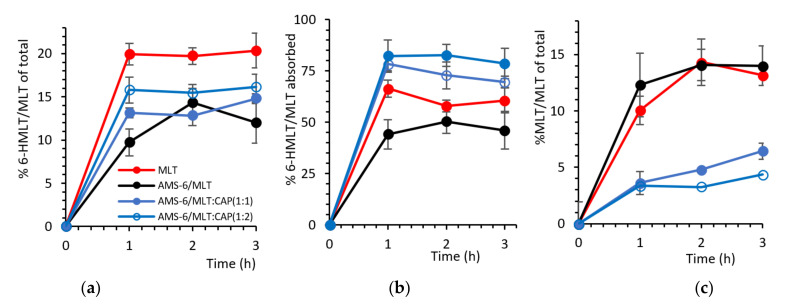
(**a**) The formation of 6-hydroxy melatonin (6-HMLT) as a molar percentage of the total MLT added on the apical side versus time. (**b**) The molar percentage of 6-HMLT as a function of the total MLT absorbed on the basolateral side. (**c**) Remaining MLT after the enzymatic reaction as a molar percentage of the total MLT added on the apical side versus time. Every data point represents the average value obtained from a triplicate sample, and the error bars correspond to the calculated standard deviation.

The percentage of MLT absorbed across the Caco-2 monolayer and metabolised into 6-hydroxy melatonin (6-HMLT) by the CYP1A2 enzyme is shown in Figure 5a, expressed as a molar percentage of the total MLT added on the apical side of the Transwell (i.e., 11.6 µg/mL of MLT). Pure MLT results in the highest conversion to 6-HMLT, with approximately 20% of MLT metabolised within 1 h. Despite AMS-6/MLT having a similar absorption rate to pure MLT in the first hour, only approximately half the percentage of total MLT is converted to 6-HMLT (Figure 5a). In the following 2 h, the conversion of MLT increases for the sample of AMS-6/MLT, but it never reaches the levels of pure MLT. At 2 and 3 h, the percentage of MLT converted into 6-HMLT is not significantly different for the samples of AMS-6/MLT, AMS-6/MLT:CAP(1:1) and (1:2). Figure 5b shows that the percentage of MLT that is converted into 6-HMLT in comparison to the MLT absorbed through the Caco-2 monolayer for pure MLT and AMS-6/MLT is different during the first hour but not after. The difference present in the first hour can be due to the dissolution of silica in the buffer, transport of silicic acid to the basolateral side [51] and the interaction of silicic acid with the hydroxylation enzyme. In the first 2 h, the percentage of MLT converted into 6-HMLT in comparison to the MLT absorbed is significantly higher for AMS-6/MLT:CAP(1:1) and (1:2) than for the other samples. The percentage of MLT still present on the basolateral side is significantly higher for pure MLT and AMS-6/MLT compared to the CAP-coated samples. The higher conversion of MLT and the lower MLT percentage in the basolateral side for the CAP-coated samples can be explained by their lower permeability (Figure 4). As the permeability rate is low, the enzyme is readily available to quickly convert MLT at any time point. Correspondingly, the amount of available MLT remaining on the basolateral side (Figure 5c) is lowest for the CAP-coated samples and highest for AMS-6/MLT and MLT alone.

## 4. Discussion

The dissolution of MLT in SGF is delayed by the coating of AMS-6/MLT with CAP, and the delay increases to over 30 min for AMS-6/MLT:CAP(1:1). These results confirm the enteric coating properties of CAP [52,53]. Previous studies demonstrated a low cytotoxicity profile in macrophage viability [54] and dendritic cells at concentrations of mesoporous silica equal to 50 µg/mL [55]. In vivo animal experiments also showed a large safety window for AMS-6 particles after oral gavage [19]. There is, thus, no reason to suspect that the concentration of AMS-6 used in our experiments would have a negative impact on the viability of the Caco-2 cells. The permeability study showed a significant decrease in permeations with the combination of AMS-6 and CAP without a disruption of the integrity of the Caco-2 monolayer. Increases in the drug permeability through the Caco-2 cell monolayer enhanced by mesoporous silica particles have been reported for other drug compounds [56,57]. This suggests that CAP is the excipient decreasing the permeation of MLT through Caco-2. Measurements of free MLT permeability across Caco-2 cell monolayers have found the permeability coefficient to be approximately 1.2 × 10^−5^ cm s^−1^ [58,59], consistent with the values obtained here and suggesting a significant decrease in the permeability of CAP-coated particles (4.81 × 10^−6^ cm s^−1^ for AMS-6/MLT:CAP 1:2). However, the results suggest that the decrease in permeability does not translate to an increase in available MLT in a Caco-2 cell model that takes into account the catabolism of MLT by CYP1A2. Rapid dissolution and release from AMS-6 leads to a higher % of available MLT on the basolateral side. In comparison to free MLT, encapsulation within AMS-6 alone results in a lower catabolism to 6-HMLT, despite similar absorption kinetics and permeability coefficients (3.08 × 10^−5^ to 2.81 × 10^−5^ cm s^−1^, respectively). After 1 h, 44.2% of the absorbed MLT was metabolized to 6-HMLT when released from AMS-6 versus 66.5% for the free MLT. Previous studies have suggested that MLT crosses the gastrointestinal barrier mainly through a transcellular pathway [18]. It has been suggested that pharmaceutical excipients may interfere with tight junctions enhancing the paracellular pathways for MLT absorption [18]. In this study, no significant decreases in resistance from the TEER measurements were observed for the AMS-6/MLT formulation in comparison to MLT alone (Figure 4c), suggesting that AMS-6 particles do not contribute to enhancing paracellular absorption through Caco-2 cells. The endocytosis of mesoporous silica particles by Caco-2 cells has been demonstrated, contributing to the enhancement of the adsorption kinetics of antibiotic drugs and drug models [60,61]. Despite not detecting a decrease in the TEER values, the ICP-MS analysis confirmed the presence of silicon in the basolateral solution of AMS-6/MLT and AMS-6/MLT:CAP(1:2). After 3 h, more than 55% of the silicon crossed the Caco-2 monolayer. It is already known that mesoporous silica can dissolve at pH between 7 and 10 [62] and that silicon crosses the Caco-2 monolayer via paracellular transport [51]. What remains to be determined is if silicon can interact with the CYP1A2 enzyme and modify its activity. Further studies at longer time intervals are required to understand the mechanism for the lower conversion of MLT to 6-HMLT in the AMS-6/MLT formulation and if the particles cross the Caco-2 cell monolayer through a transcellular pathway, inhibiting the activity of the CYP1A2 enzyme.

## 5. Conclusions

Overall, mesoporous AMS-6 particles loaded with MLT and coated with cellulose acetate phthalate significantly altered the kinetic profile of MLT, resulting in a delayed release under simulated gastric conditions, with a suppression of dissolution of up to 40 min at high cellulose coating ratios. This translated into a lower permeability coefficient in the Caco-2 cell monolayers for the cellulose-coated AMS-6 particles (1.06 × 10^−5^ and 4.81 × 10^−6^ cm s^−1^ for AMS-6/MLT:CAP(1:1) and (1:2), respectively), with a complementary effect of the silica mesopores and the cellulose coating in slowing the absorption kinetics. The different formulations studied did not affect the tight junctions or integrity of the cell monolayers. The slower absorption kinetics of the cellulose-coated particles resulted in a higher catabolism of MLT by the CYP1A2 enzyme into 6-hydroxy melatonin and a decrease in the amount of available melatonin. The levels of MLT were also high for the AMS-6 formulation in the absence of a coating, due to a lower catabolism of the hormone by the CYP1A2 enzyme. The presence of silicon on the basolateral side suggests a dissolution of the silica and transport of silicon across the Caco-2 cells monolayer. Further work is required to determine if silicon interacts with CYP1A2, inhibiting its activity. The experiments will also focus on testing these formulations in animal models to determine the effects of the mesoporous silica and CAP coating on the pharmacokinetics of melatonin and explore the potential effects on the sleep/wake cycle.

## Figures and Tables

**Table 1:** Kinetic release constants for the AMS-6/MLT and AMS-6/MLT:CAP formulations for the release curves in SGF (pH 1.2) and SIF (pH 6.8). * First-order release kinetics. ** Zero-order release kinetics was applied, assumed for MLT release in SGF and SIF, respectively.

Media	Formulation	K_H_	R^2^	t50 min
SIF	MLT **	2.547	0.989	20
	AMS-6/MLT	9.759	0.980	30
	AMS-6/MLT: CAP(1:1)	14.271	0.968	15
	AMS-6/MLT:CAP(1:2)	14.511	0.992	15
SGF	MLT *	0.017	0.952	60
	AMS-6/MLT	5.376	0.915	135
	AMS-6/MLT:CAP(1:1)	3.197	0.997	270
	AMS-6/MLT:CAP(1:2)	3.108	0.992	330

## Data Availability

All data supporting research results presented are available upon request to the corresponding author.

## Supplementary Materials

The following are available online at https://www.mdpi.com/article/10.3390/pharmaceutics13091436/s1, Figure S1: DLS hydrodynamic particle size distribution of calcined AMS-6 particles in water and FTIR spectra of MLT and the formulations. Figure S2: Kinetic release measurements of MLT and the test formulations in SIF and SGF.

## Abbreviations

6-HMLT	6-Hydroxymelatonin
AMS-6	Anionic surfactant templated mesoporous silica
APES	(3-Aminopropyl)triethoxysilane
CAP	Cellulose acetate phthalate
CYP	Cytochrome P450 enzyme
DMEM	Dulbecco’s modified Eagle’s medium
DMSO	Dimethyl sulfoxide
DSC	Differential scanning calorimetry
FBS	Foetal bovine serum
FT-IR	Fourier-Transform Infrared spectroscopy
GI	Gastrointestinal
HBSS	Hank’s balanced salt solution
HEPES	4-(2-Hydroxyethyl) piperazine-1-ethane sulfonic acid
HPLC	High-performance liquid chromatography
MLT	Melatonin
PBS	Phosphate-buffered saline
PEST	Penicillin-streptomycin solution
RT	Room temperature
SEM	Scanning electron microscopy
SGF	Simulated gastric fluid
SIF	Simulated intestinal fluid
TEER	Transepithelial/transendothelial electrical resistance
TEM	Transmission electron microscopy
TEOS	Tetraethyl orthosilicate
TGA	Thermogravimetric analysis
Tmax	time to reach maximal concentration
XRD	X-rays diffraction.
