# Supplementary material for: Effects of Absorption Kinetics on the Catabolism of Melatonin Released from CAP-Coated Mesoporous Silica Drug Delivery Vehicles
Source: Pharmaceutics. 2021 Sep 9;13(9):1436. doi: 10.3390/pharmaceutics13091436 (PMC8464897; doi:10.3390/pharmaceutics13091436)
Supplement: Supplementary file 1 [file pharmaceutics-13-01436-s001.zip › pharmaceutics-1364054-supplementary.pdf]

pharmaceutics

MDPI

Supplementary Materials: Effects of Absorption Kinetics on the Catabolism of Melatonin Released from CAP-Coated Mesoporous Silica Drug Delivery Vehicles

Irene Moroni and Alfonso Garcia-Bennett

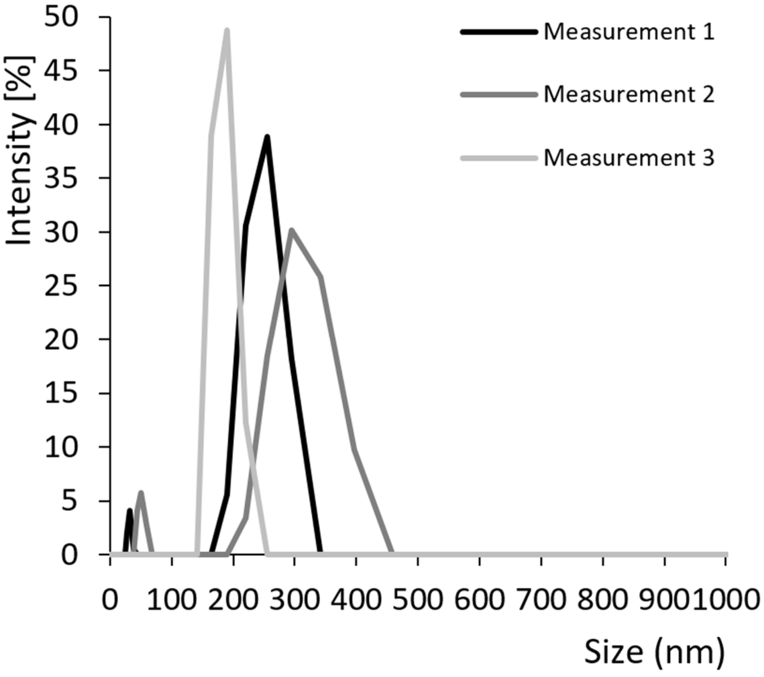

(**a**)

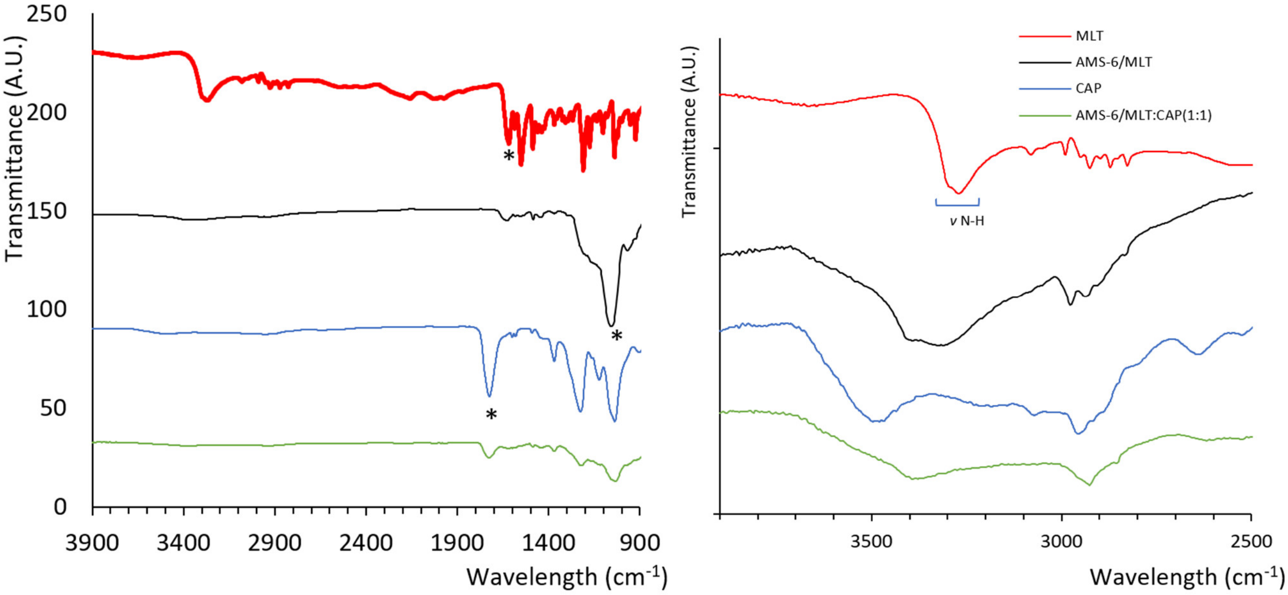

(**b**)

Figure S1. (**a**) DLS hydrodynamic particle size distribution of calcined AMS-6 particles in water, with an average value of 246.7 ± 62.6 nm. (**b**) FT-IR spectra of MLT and formulations prepared in this manuscript. Asterisk's mark stretching frequencies for signature bonds in each spectrum. An enlarged area of the region between 2000-4000 cm^{-1} is shown on the left.

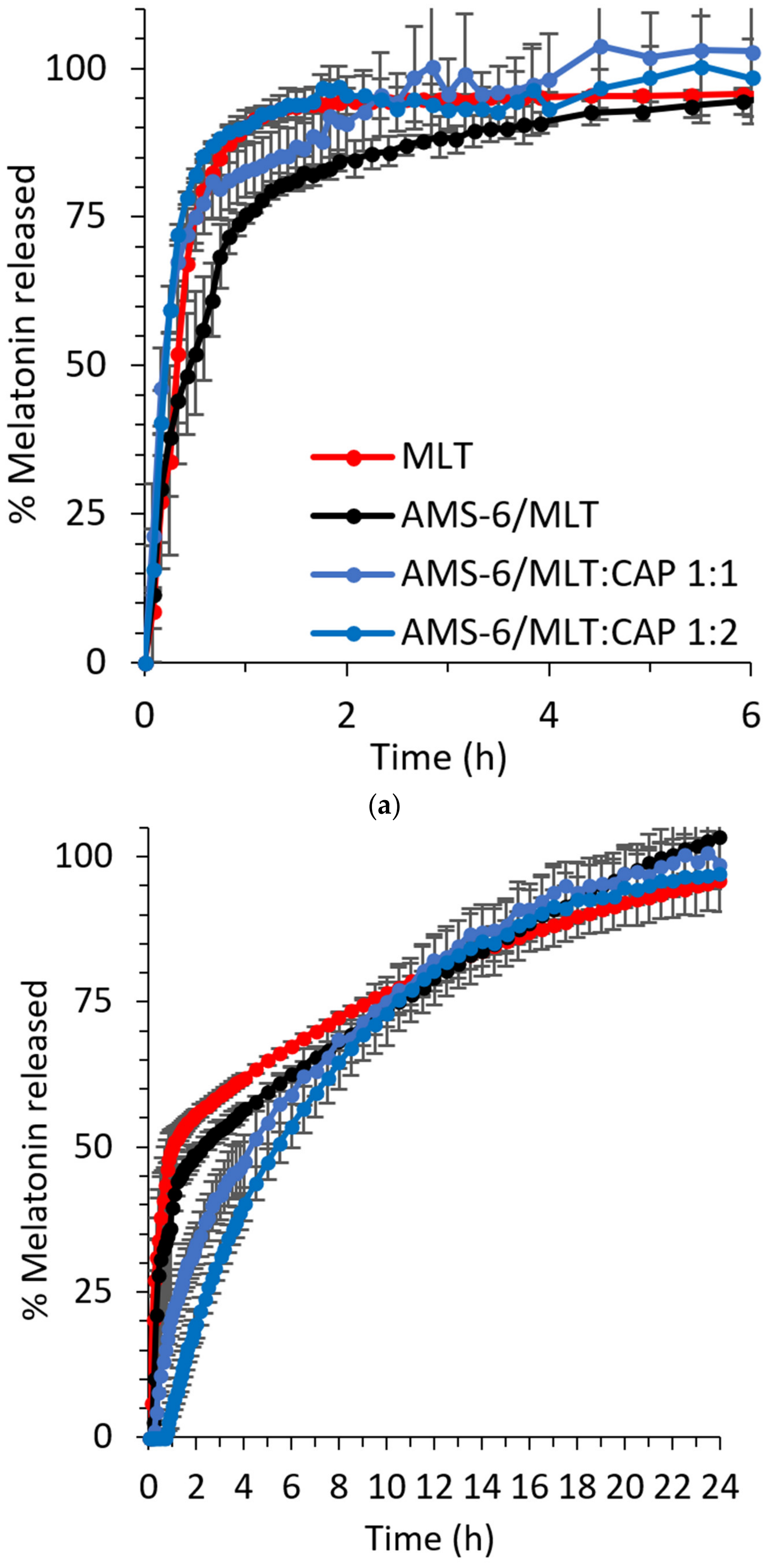

(**a**)

(**b**)

Figure S2. (**a**) Kinetic release measurements of MLT and test formulations in SIF (pH 6.8) and (**b**) SGF (pH 1.2).
